# Mobile Health Intervention to Increase Oral Cancer Therapy Adherence in Patients With Chronic Myeloid Leukemia (The REMIND System): Clinical Feasibility and Acceptability Assessment

**DOI:** 10.2196/mhealth.8349

**Published:** 2017-12-06

**Authors:** Amanda Pereira-Salgado, Jennifer A Westwood, Lahiru Russell, Anna Ugalde, Bronwen Ortlepp, John F Seymour, Phyllis Butow, Lawrence Cavedon, Kevin Ong, Sanchia Aranda, Sibilah Breen, Suzanne Kirsa, Andrew Dunlevie, Penelope Schofield

**Affiliations:** ^1^ Centre for Nursing Research Cabrini Institute Malvern, Victoria Australia; ^2^ Faculty of Medicine, Nursing and Health Sciences Monash University Clayton, Victoria Australia; ^3^ Department of Cancer Experiences Research Peter MacCallum Cancer Centre East Melbourne, Victoria Australia; ^4^ School of Nursing and Midwifery Faculty of Health Deakin University Geelong, Victoria Australia; ^5^ Department of Haematology Royal Adelaide Hospital Adelaide, South Australia Australia; ^6^ Department of Haematology Peter MacCallum Cancer Centre East Melbourne, Victoria Australia; ^7^ Sir Peter MacCallum Department of Oncology Faculty of Medicine, Dentistry and Health Sciences The University of Melbourne Parkville, Victoria Australia; ^8^ School of Psychology The University of Sydney Sydney, New South Wales Australia; ^9^ School of Science RMIT University Melbourne, Victoria Australia; ^10^ Cancer Council Australia Sydney, New South Wales Australia; ^11^ Public Health Group, Stroke Division The Florey Institute of Neuroscience and Mental Health Heidelberg , Victoria Australia; ^12^ Pharmacy Department Monash Health Clayton, Victoria Australia; ^13^ Faculty of Pharmacy and Pharmaceutical Sciences Monash University Parkville, Victoria Australia; ^14^ Department of Psychology School of Health Sciences, Faculty of Health, Arts and Design Swinburne University of Technology Hawthorn, Victoria Australia

**Keywords:** mobile phone, neoplasms, Internet, medication adherence

## Abstract

**Background:**

Optimal dosing of oral tyrosine kinase inhibitor therapy is critical to treatment success and survival of patients with chronic myeloid leukemia (CML). Drug intolerance secondary to toxicities and nonadherence are significant factors in treatment failure.

**Objective:**

The objective of this study was to develop and pilot-test the clinical feasibility and acceptability of a mobile health system (REMIND) to increase oral drug adherence and patient symptom self-management among people with CML (chronic phase).

**Methods:**

A multifaceted intervention was iteratively developed using the intervention development framework by Schofield and Chambers, consisting of defining the patient problem and iteratively refining the intervention. The clinical feasibility and acceptability were examined via patient and intervention nurse interviews, which were audiotaped, transcribed, and deductively content analyzed.

**Results:**

The intervention comprised 2 synergistically operating elements: (1) daily medication reminders and routine assessment of side effects with evidence-based self-care advice delivered in real time and (2) question prompt list (QPL) questions and routinely collected individual patient adherence and side effect profile data used to shape nurses’ consultations, which employed motivational interviewing to support adoption of self-management behaviors. A total of 4 consultations and daily alerts and advice were delivered over 10 weeks. In total, 58% (10/17) of patients and 2 nurses participated in the pilot study. Patients reported several benefits of the intervention: help in establishing medication routines, resolution of symptom uncertainty, increased awareness of self-care, and informed decision making. Nurses also endorsed the intervention: it assisted in establishing pill-taking routines and patients developing effective solutions to adherence challenges.

**Conclusions:**

The REMIND system with nurse support was usable and acceptable to both patients and nurses. It has the potential to improve adherence and side-effect management and should be further evaluated.

## Introduction

Chronic myeloid leukemia (CML) is an uncommon clonal bone marrow stem cell disorder. The disease has a triphasic natural history—commencing in chronic phase, unless well controlled, progressing to accelerated phase, and ultimately to blastic phase with over 90% of patients being diagnosed in the chronic phase [1]. Oral tyrosine kinase inhibitor (TKI) therapy is the standard of care for patients with chronic-phase CML. Imatinib, nilotinib, and dasatinib, currently first-line oral TKI therapies, are highly successful in improving progression-free and overall survival [2-4]. However, optimal adherence to TKIs is critical to treatment success and survival, with continuous, daily dosing required for an indefinite period, often lifelong [4,5], unless complete deep molecular response has been achieved when a treatment-free period can be tested [6,7].

TKIs are associated with numerous potential toxicities, including myelosuppression, nausea, diarrhea, fatigue, and soft-tissue edema, especially in the face and lower legs [8-11]. Given these toxicities, it is not surprising that medication adherence is problematic; a recent review found that one-third to one-quarter of patients with CML have poor TKI adherence [12]. This is serious as treatment response is compromised for patients with less than 90% adherence [13,14]. Greater frequency of adverse events and higher levels of patient-reported symptoms predict lower levels of medication adherence [15,16]. Conversely, good knowledge of disease and treatment and confidence in medication self-management are linked to improved adherence [15,17], whereas forgetfulness is the most common reason for unintentional nonadherence [18]. These factors are potentially modifiable by educational and behavioral interventions. There is an urgent need to improve patient medication adherence among patients with CML [4,19,20].

Mobile health (mHealth), defined as employing mobile devices to support medical practice and public health, may improve TKI adherence using cellular phone apps and text messages. Internationally, phone text interventions have been tested to prompt oral drug adherence in a number of chronic conditions, including human immunodeficiency virus/acquired immunodeficiency syndrome (HIV/AIDS) [21-27], diabetes mellitus [28-31], asthma [32], and tuberculosis [33,34]. Studies reported short- [21,23,24,28,29,32] and long-term [26,29] improvements in adherence, with patients generally perceiving text reminders as beneficial [21,22,27,33,34]. Reviews of phone text reminder interventions have demonstrated efficacy in enhancing patients’ compliance to drug therapy [35-39]; however, only one study of cancer patients was identified, which was targeted at adolescents and young adults and phone text messages were not utilized [40]. A secondary in-depth analysis found no published trials that investigated a medication adherence intervention that integrates nurse-led phone consultations with mHealth systems in oncology [41].

**Figure 1:**
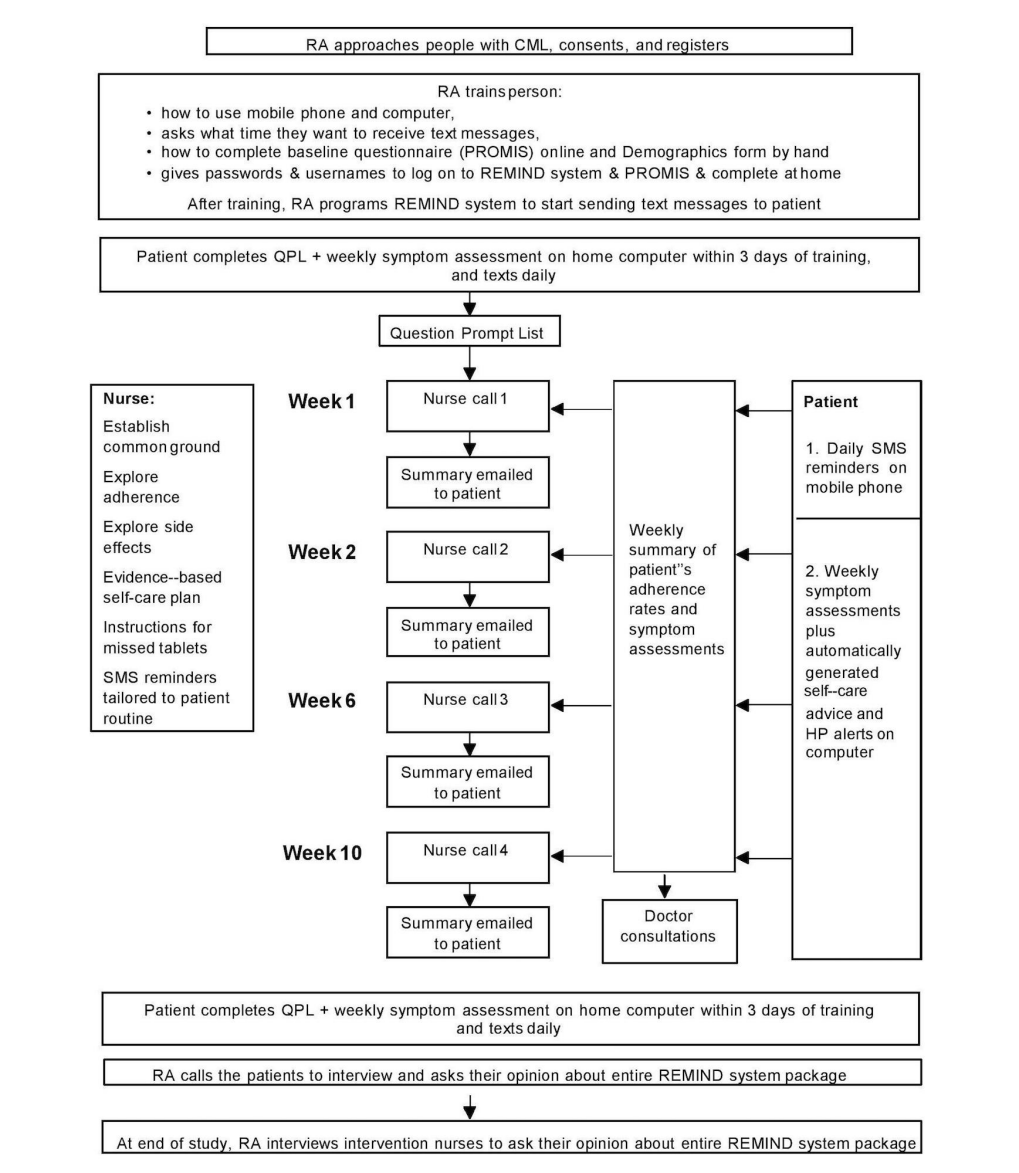
Flowchart of the nurse-mediated telehealth (including REMIND system) intervention package (CML, chronic myeloid leukaemia; HP, health professional; QPL, question prompt list; RA, research assistant; SMS, short message service).

Oncology nurses are central to providing education and coaching in patient self-management of drug toxicities and medication adherence [16,42,43]. Patient and clinician acceptability of nurse-led mHealth interventions aimed at improving postchemotherapy symptom management has been demonstrated in solid tumors [44,45]. We conceived a multifaceted intervention package consisting of 2 integrated elements delivered over 10 weeks: (1) the REMIND mHealth system comprising daily medication reminder texts and individualized self-care advice based on self-reported side effects delivered in real time and (2) nurse telephone consultations to promote adherence to imatinib (Glivec) and coach patients in toxicity self-management (Figure 1). Briefly, after the patient was trained in using the REMIND system, he or she completed a Web-based symptom survey and question prompt list (QPL), which was used to guide the first nurse telephone consultation. Daily, the patient was asked to respond to a text reminder message for each dose based on his or her individual regimen.Once a week, a text message was sent to patients to remind them to complete an online symptom survey. For moderate to severe symptoms, self-care information was sent to the patient in real time.

Adherence and symptom profiles were available for clinician review and used to guide 3 more nurse telephone consultation. The aim of this paper is to describe the development of this intervention and its clinical feasibility and acceptability. The study is reported in accordance with the CONSORT eHealth checklist (V1.6.1) [46].

## Methods

### Phase 1: Development of the Intervention

The framework for the development of effective, clinically feasible, and sustainable interventions by Schofield and Chambers was used to guide intervention development [47]. This intervention framework represents an expansion of the Medical Research Council (MRC) framework for complex interventions development [48]. The MRC framework provides broad recommendations for intervention development, such as establishing the theoretical and evidence base and testing procedures. The intervention development framework by Schofield and Chambers specifies 7 features: (1) targeting cancer type and stage, (2) tailoring to unique individual needs, (3) promoting self-management, (4) efficient intervention delivery, (5) ensuring evidence-based and theoretical grounding, (6) specifying protocol training and adherence, and (7) confirming stakeholder acceptability.

The features of the framework by Schofield and Chambers [47] used to guide intervention development are described below.

#### Targeting

To understand the problem, a prior qualitative study by this team with 16 patients with CML prescribed a TKI and 10 health professionals was conducted to examine the nature, extent, and reasons for medication nonadherence [49]. Findings revealed that nonadherence was reported on at least one occasion among 75% of patients sampled with reasons for unintentional nonadherence including forgetfulness or misunderstanding medical instructions. Intentional nonadherence was related to side effects and insufficient health care support. Health professionals also experienced difficulty in accurately evaluating the medication adherence of their patients. These findings, in addition to those of others [17], indicate that intervention goals should be to increase depth of knowledge of disease, treatment regimen, and toxicities; increase confidence in self-management of side effects; and prompt TKI self-administration according to medical advice in relation to timing, dose, and frequency.

#### Tailoring

For the REMIND system, the timing of daily text-based medication reminders could be set to suit individual preferences. The evidence-based self-care advice was electronically delivered in real time, corresponding to the individual’s responses to weekly self-assessment of side effects. The structure and content of the nurse consultations were directed by a QPL administered electronically to the patient just before the first nurse consultation.

#### Promoting Self-Management

Successful self-management requires the ability to self-assess problems; marshal information, skills, or resources to problem-solve; set goals; and implement the planned solution. Key to this is self-efficacy, which is defined as a person’s beliefs in his or her ability to succeed in a given task. Although the REMIND system (medication reminders texts, side-effect assessment, and self-care advice) facilitated self-assessment of side effects and provided information and resources, coaching in self-management and behavior change was accomplished by the nurse-led phone consultations using motivational interviewing, which is a client-centered method that allows patients to explore and resolve their own ambivalence about adopting a new behavior, such as medication adherence, and to evoke self-motivation as opposed to traditional didactic health advice provision [50].

#### Efficiency

The combined delivery methods of this intervention were used to increase the efficiency of the intervention. Blending nurse phone consultations with the REMIND system increased the dose of the intervention, permitting the nurses to focus their expertise on coaching. Exclusive use of communication technologies for intervention delivery was intended to increase access to those living in rural communities or those who were too ill or had less time to attend face-to-face consultations.

#### Evidence and Theory

Both intervention content and delivery mechanism should be based on theory and available evidence. The list of relevant drug toxicities was generated using Monthly Index of Medical Specialties and expert clinician advice (JFS). Authors (PS and SA) updated their systematic review of self-care strategies for chemotherapy side effects [51] with additional literature searches to develop self-care recommendations for imatinib side effects in conjunction with an expert clinician (JFS). A consumer with CML (AD) iterative reviewed all content along with the multidisciplinary study team. Motivational interviewing, the central technique used the nurse delivery of content, arose from self-determination theory [52], which is a framework of intrinsic and extrinsic motives that facilitate or forestall behavior change. Motivational interviewing by telephone is significantly associated with improving medication adherence [53].

#### Protocolization

Interventions that layer digital technologies with targeted clinician contact rely on adequate training and supervision to ensure uniform delivery of content across intervention clinicians. Standardized manuals were developed specifying (1) the intervention content, including the resource manual describing the disease, treatments, side effects, and evidence-based, self-management advice; and (2) the training and supervision protocols. Protocolization of standardized content and training ensures a comprehensive knowledge base and attainment of core skills required by the intervention nurses and consistent, standardized, and reproducible delivery of the intervention content.

#### Stakeholder Acceptability

Using a codesign process involving the end users (clinicians and patients) throughout the development process optimizes stakeholder acceptability [54,55]. Our stakeholder qualitative research [49] directed the intervention goals and structure to ensure relevance. A clinical nurse specialist was engaged to write the nurse intervention manuals, detailing the content of each intervention session. A resource manual to support the provision of evidence-based advice was developed from booklets, fact sheets, and websites which were sourced from reputable peak cancer and other health bodies, in particular, the Leukaemia Foundation of Australia. The intervention development working party consisting of a consumer, hematologists, hematology nurse specialists, clinical psychologists, behavioral scientists, and an oncology pharmacist reviewed iterative revisions of intervention and resource manuals.

### Phase 2: Pilot Testing

#### Setting

This study was conducted in a comprehensive cancer hospital in Melbourne, Australia.

#### Design

A qualitative research design was used to examine the clinical feasibility and acceptability of the intervention package to patients and nurses.

#### Participants

Participants were patients who received the intervention and nurses who delivered the intervention. Inclusion criteria for patients were as follows: age >18 years, proficiency in English, a confirmed diagnosis of chronic-phase CML with no signs of progression, and >3 months of continuous treatment with imatinib (Glivec) with no evidence of drug resistance. On the basis of feedback from the first 3 patients in this pilot testing phase, this last criterion was changed to treatment with imatinib and no evidence of drug resistance. Exclusion criteria were cognitive or psychological difficulties as assessed by the patient’s treatment team and being too unwell. In total, 2 clinical nurse consultants with postgraduate qualifications in cancer nursing and extensive experience in hematological oncology were trained to deliver the intervention.

#### Nurse Training

The nurses were trained to deliver the structured telephone consultations and use the REMIND system in a 1-day workshop, facilitated by a clinical communication expert (PS). The workshop comprised an overview of the project and intervention manual and training in (1) identifying and responding to emotional cues on the phone, (2) exploring concerns about medication adherence and providing evidence-based self-care advice, and (3) coaching patients using motivational interviewing techniques. Didactic instruction was complemented by 2 role-play sessions with a simulated patient and facilitator feedback: 1 in the workshop and 1 in a subsequent phone consultation, which was audiorecorded for self-appraisal and facilitator feedback.

#### Procedure

Potentially eligible participants were identified by clinician referral or pharmacy records of imatinib dispensing and examined for eligibility as per the eligibility criteria by the research assistant. After confirming eligibility and suitability with the treating clinician, patients were approached either face-to-face at outpatient clinic visits or over the telephone. An appointment was made with interested participants to review and sign the consent form, complete a baseline questionnaire, and be trained to use the REMIND system. All patients then received the intervention over 10 weeks and at the end of the intervention, completed a follow-up questionnaire. The patient-reported outcomes in both questionnaires covered quality of life [56], psychological morbidity [57], and self-management [58].

#### Patient and Intervention Nurse Interviews

Following completion of the intervention, consenting patients and intervention nurses participated in a semi-structured telephone interview of 20 to 40 min. Separate interview schedules were developed for patients and health professionals. Both interview schedules covered perceptions of the intervention package, including content, timing, and perceived utility of each component of the REMIND system and the nurse consultations, perceived impact of the intervention on adherence and self-management, overall satisfaction with intervention, and recommended changes. All interviews were audiorecorded and transcribed verbatim.

A content analysis was undertaken to identify themes [59]; 2 investigators (APS and JW) analyzed intervention patient data and 2 investigators (PS and JW) analyzed nurse data separately. The process involved iterative movement between transcripts, rereading to establish familiarity with content and reflection of ideas. Similar content was collated and summarized to form themes with codes assigned by each analyst. Following analysis completion, all data analysts met to discuss results of their respective data. Disagreements between coders were identified and discussed until agreement was reached. Coding themes were then agreed upon, and the data from each group were coded into these themes. Themes were then collapsed or divided through consultation and discussion. As the interviews covered similar topics, perspectives of the 2 groups were aggregated. Survey outcomes were not analyzed due to sample size.

## Results

### Phase 1: Development of the Intervention

#### The REMIND System

**Figure 2:**
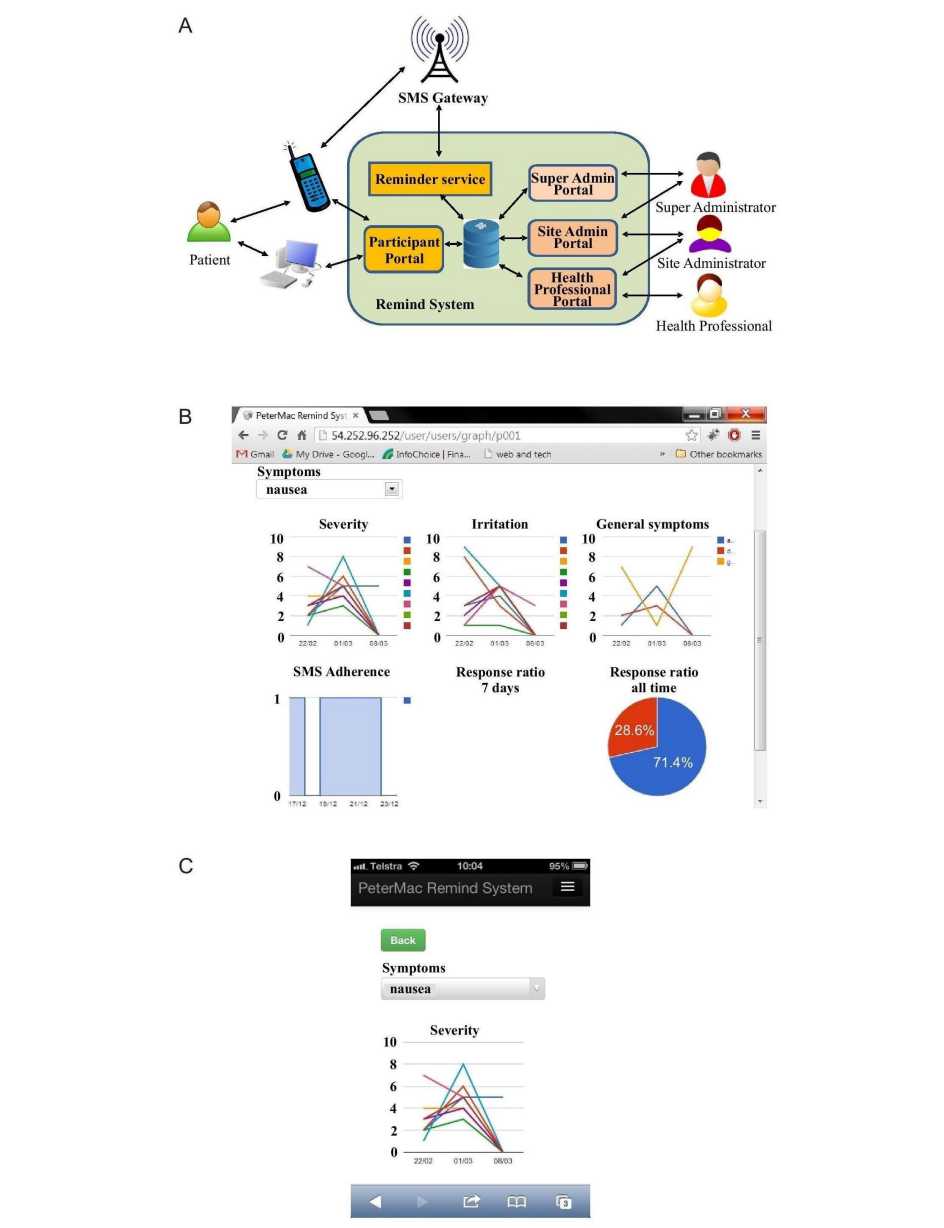
REMIND system representation. (A) Architecture of the REMIND system showing context of interaction with different types of users (patients, health care professionals, and administrators) and external SMS Gateway service. The patient receives reminders on his or her cellular phone, sent from the SMS Gateway, and can interact with the weekly symptoms survey via a browser on a standard computer or a smartphone. (B) Screenshot of patient dashboard, showing graphs (from left to right) of scores (for severity and irritation) for symptom (in this case, nausea) over time, bar chart indicating daily adherence (measured by response), and pie chart showing overall adherence rate. (C) Screenshot of specific symptoms—severity graph for nausea—as displayed on the cellular smartphone interface.

The REMIND system was a hosted Web application, deployed on the Amazon Web Services cloud platform as an EC2 instance, with multiple Web- and text-based interfaces provided for a variety of users and a range of services that control text messages sent to a third-party short message service (SMS) text messaging Gateway (Figure 2). It comprises the following:

1. A database of all user information, including patient and health professionals’ log-in information; patient phone numbers and preferred notification time for medication reminders; and a log of all responses to daily reminders and answers to weekly survey questions
2. A Web-based application that allows patients and health professionals to view, and for health professionals to edit information regarding users, and patients to edit responses to reminders and symptom survey answers. The pages displayed by this application are designed for both laptop/desktop and mobile phone screens.
3. An automatic nightly service that pulls responses from the SMS Gateway, updates the database, creates SMS messages for the next day from information in the user database, and then forwards these messages to the SMS Gateway to be queued to be sent out at the preferred notification times. This program is sensitive to time zone and daylight saving and automatically adjusts the send-time of messages according to the local time of the patient’s location.

Daily or twice-daily cellular phone reminder message were sent via the REMIND system to each patient for each dose based on patients’ individual dosing regimen. Patients were asked to reply with a *Yes* message once the dose was taken or *No* if not taken. Adherence, patient compliance with imatinib, was considered to be a response of *Yes* received via a phone message from patients. If no reply was received within a designated time period (2 hours), this was interpreted as *No*.

Once a week, patients completed a side-effect assessment online via the REMIND system using a *smart* cellular phone or computer. For each of 11 potential side effects/symptoms patients may experience when taking imatinib, they reported severity and extent of bother on a scale of 1-10 for each side effect present. Automatically generated self-care advice was customized to the symptoms reported and sent to the patients in real time.

If 10% of scheduled doses in a week were missed or if side-effect severity was ≥4, this was included on a *daily digest*, which was forwarded to the nurse assigned to that patient via email. The nurse could decide to contact the patient outside of the scheduled consultations to discuss self-management strategies or address medication adherence barrier(s).

#### Phone-Based Nurse Consultation Sessions (4 Sessions in Weeks 1, 2, 6, and 10)

In the week before the first nurse consultation, the patient reported medication adherence daily, completed the weekly medication side-effect assessment, and selected questions from the CML-specific QPL. QPLs are structured list of commonly asked questions customized to disease type and have been found to improve clinician-patient communication and increase the amount of desired information received by patients [60]. The nurse accessed the REMIND system to view the patient’s responses to shape content of the first nurse consultation session. It covered the following: (1) building rapport; (2) addressing questions noted on the QPL; (3) establishing *common ground* by assessing patients’ understanding of their diagnosis, current symptom experience, and medication regimen; (4) discussing the patient’s medication adherence and explored adherence barriers; (5) coaching to address barriers; (6) discussing medication side effects; (7) coaching in evidence-based self-care strategies targeting each reported side effect; and (8) summarizing the consultation and assessing patient understanding. The subsequent consultations used the REMIND system’s patient-reported information to cover (4) to (8).

### Phase 2: Pilot Testing

**Figure 3:**
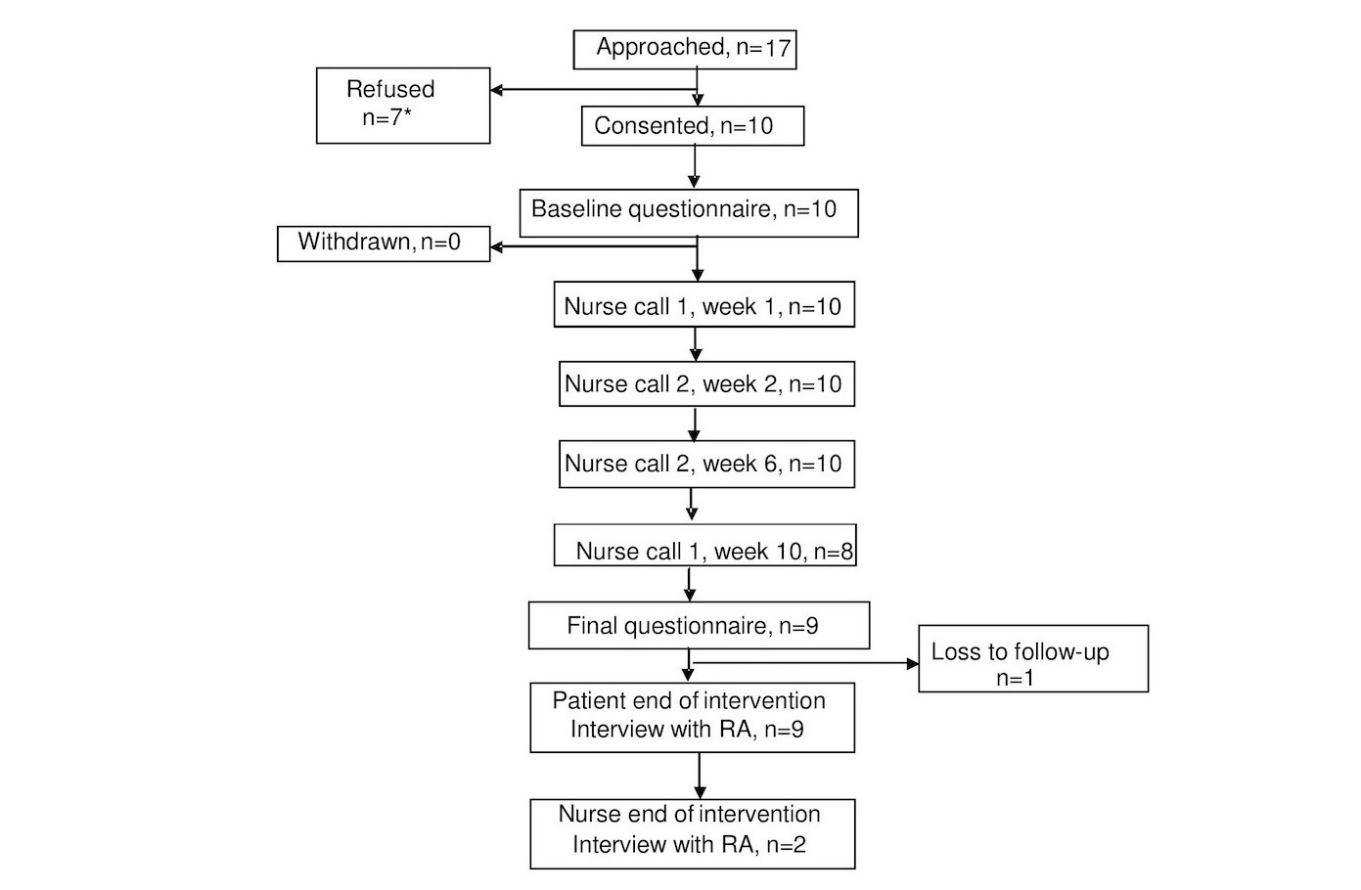
CONSORT flowchart of participants completing each intervention component and data collection point (RA refers to research assistant).

A total of 10 out of 17 patients agreed to participate, with a response rate of 58% (Figure 3). Of the participants, 9 completed all components of the intervention and 1 participant ceased before week 10 due to travel. Another participant was lost to follow-up. In total, 9 patients and 2 nurses completed interviews.

Participant demographics are described in Table 1. Median age of patient participants was 54 years, ranging from 35 to 72 years; the majority of participants, that is, 6 out of 9, were male (67%). The 9 patients who participated in the pilot testing had received imatinib therapy for a median of 4 years before the start of the study, with a range from 15 days to 12 years.

**Table 1:** Participant demographics.

Characteristics		Patients (N=9)
**Age (years)**		
	Median (interquartile range)	54 (44.5-60.0)
	Range	35-72
**Sex, n (%)**		
	Male	6 (67)
	Female	3 (33)
**Country of birth, n (%)**		
	Australia	7 (78)
	China	1 (11)
	England	1 (11)
**English as first language, n (%)**		
	Yes	8 (89)
	No	1 (11)
**Education (highest level completed), n (%)**		
	Secondary/high school	3 (33)
	Trade/Technical and Further Education	1 (11)
	Bachelor’s degree	3 (33)
	Postgrad diploma/masters/PhD	2 (22)
**Time since diagnosis**		
	Median (interquartile range in years)	4 (1-13)
	Range	1 month-17 years
**Imatinib treatment duration**^a^		
	Median (interquartile range in months)	48 (16-123)
	Range	15 days-12 years
**Employment status, n (%)**		
	Full time	4 (44)
	Part time	2 (22)
	Home duties	1 (11)
	Retired	2 (22)
**Residence**		
	Metropolitan	4 (44)
	Rural	5 (56)

^a^Duration on imatinib at the start of REMIND study: Patient 2 (9 years), Patient 3 (5 years), Patient 4 (4 years), Patient 5 (2.5 years), Patient 6 (11.5 years), Patient 7 (1 year), Patient 8 (1.6 years), Patient 9 (12 years), Patient 10 (2 weeks).

The system administrator as detected by the REMIND system monitored adherence (Table 2). The SMSs were sent on time (<1 hour) to patients on 671 out of 684 (98.0%) occasions, with a range of 0-7% participants experiencing SMS failure and a range of 0-22% participants not replying to SMS messages at all, over the total study period. In total, 2 patients reported that up to 40% of messages were not received by them by the appointed time due to problems with slow networks, particularly in rural areas.

A number of common themes were identified across the nurses and patients.

**Table 2:** System administrator details of SMS (short message service) texts sent and answered by patients.

Patient	Total days	SMS failure, n (%)	Patient failure to answer SMS when sent, n (%)	
			In 2 hours of SMS	Not at all
2	74	5 (7)	5 (7)	5 (7)
3	79	1 (1)	3 (4)	3 (4)
4	62	0 (0)	4 (7)	3 (5)
5	69	0 (0)	14 (20)	10 (15)
6	120	0 (0)	8 (7)	6 (5)
7	79	0 (0)	4 (5)	4 (5)
8	80	1 (1)	32 (41)	17 (22)
9	57	0 (0)	2 (4)	0 (0)
10	75	4 (5)	8 (11)	8 (11)

#### Accountability and Routine

Patients and nurses reported that they found the intervention acceptable and helpful for improving adherence through enabling patient accountability. A newly diagnosed patient stated:

...it definitely helped reinforce habit when I was at the start of getting on, getting this medication into routine.

PT10

Longer-term patients with changed circumstances also benefited:

I’ve had overseas visitors for the last 2 weeks so I’m out of my usual routine…I’m sure I would have missed some of my drug taking…had I not had the reminders.

PT6

A nurse spoke about the intervention for modifying adherence behavior:

...we discussed the barriers that she had to taking her tablet regularly. And then over time she softened and realized that she could develop strategies to overcome the problems...

N2

#### Alleviating Psychological and Symptom Distress

Patients spoke about the benefits for alleviating psychological and symptom distress. The intervention was reported to be particularly useful for easing uncertainty about symptoms/side effects. One of the participants said:

It was good...if someone (nurse) noticed a change in symptoms I might not have picked up on it, and they were able to discuss it with me and find out why it might have happened.

PT3

Patients felt reassured by the intervention:

...the good part...was the personal touch...it’s just that really nice reminder that there’s someone out there that’s half looking out for you.

PT5

Access to expert advice was also valued.

You do talk about things with your partner but you don’t go down some of the tracks that you probably would with a nurse...they prompt information out of you from their knowledge.

PT5

#### Supporting Self-Efficacy

The intervention was identified as useful for uptake of self-management strategies and encouraging informed decision making. One patient commented:

Because I’ve been doing this, it makes me think about other things like diet.

PT2

Another patient explained self-care options and informed choice:

...we did discuss that I could take Imodium (diarrhoea medication) or something like that if I wanted to but...I didn’t want to take any more tablets...where I can’t get to a toilet and if I did need, like a wedding, I’d take Imodium to make sure that I didn’t have any accidents.

PT3

#### Functionality of Intervention

Overall, patients found the REMIND system highly usable. Although a patient (PT6) described the process as “pretty straightforward,” another mentioned:

I suppose the only bit I found a bit off-putting the most was the questions around depression and anxiety.

PT10

Patients routinely had access to a cellular phone; however, one patient admitted that they:

...struggled with that [the 2 hour window to respond].

PT 7

Most patients found the system reliable and received the messages at the appointed time, but there were exceptions:

There were maybe three times that the computer system either didn’t send a text or it was an hour or 2 late...

PT3

A rural patient had ongoing problems as he had an unreliable telephone network, and reported as many as 40% of the text messages delayed by 30-45 min [PT10]. The 2 participating nurses differed in their opinion about the REMIND system: one found it easy but the other needed time to become familiar with it. Both nurses found the initial QPL useful, comprehensive, and helpful in shaping the consultation content and facilitating rapport. The resource and intervention manuals supplied to the nurses were endorsed as comprehensive and very helpful, particularly the session checklist. Reorganization of the manual was suggested:

...I find these manuals just a bit fiddly to work around so that’s why I’ve got like post it notes everywhere...it’d be good if there was tabs or something...

N1

Training in motivational interviewing was perceived as beneficial:

I gained skills in the motivational interviewing techniques which I continue to use.

N2

Both nurses felt that the timing and number of interview sessions were appropriate with no recommendations for alterations. Both suggested that daily digest (or summary) of patient adherence and symptom responses should be emailed less frequently than daily because it was “too much.”

Overall, 3 patients fell below 90% reported adherence levels. The protocol stated patients with adherence below 90% should have been contacted outside to the scheduled calls. However, both nurses expressed that they were in a quandary when patients did not SMS *Yes*, but relied on their experience and decided not to make extra contact. One of the nurses commented:

My personal experiences on the phone calls has been that patients report that they are adhering to their medications even if they’re not responding to their SMS, so it’s kind of a bit tricky...I guess it’s a guide as to whether they’re taking their tablet but it’s not definitive...

N1

#### Optimal Intervention Target Group

In terms of timing, most patients thought the intervention would be most beneficial at the start of therapy or, even at diagnosis:

...if they’re just starting out on Glivec I reckon it would be invaluable.

PT2

The newly diagnosed participant (PT10) commented that it was “good timing” for him. Nurses agreed suggesting:

...A lot of people said it would have been helpful when they were first diagnosed.

N1

However, they also suggested that the intervention has broad applicability:

Not only in patients with CML but...there’s a whole lot of new oral medications on the market now for a whole variety of cancers and conditions.

N1

## Discussion

### Principal Findings: Intervention Development

The framework by Schofield and Chambers was successfully applied to the development process. Preliminary qualitative research [49] served to delineate the patient problem and aligned the intervention goals with patients’ needs. Relevant evidence and theoretical perspectives were investigated through literature reviews and combined with reputable patient education material to create appropriate evidence-based content and resources for the intervention. The automated features of the REMIND system permitted flexibility in intervention content and responsiveness to individualized concerns. Combining automated reminders, side-effect assessments, and self-care advice with personalized coaching fostered self-management. The *dose* of the intervention was maximized by using resource-intensive nursing expertise efficiently by basing the content of consultations on automated reminders and symptom assessments. While the standardized material supports reproducibility, the codesign processes promote acceptability. These approaches are consistent with the current literature that recognizes improved knowledge of disease course, medication, and management of side effects paired with expert clinician support as integral to improving medication adherence in patients with CML [15].

### Principal Findings: Acceptability and Clinical Feasibility of Intervention

Pilot testing demonstrated that this intervention was able to be implemented and integrated into the clinical management of 10 patients with CML. It was highly acceptable to both patients and nurses. Most patients indicated that receiving and responding to the text reminders prompted medication adherence due to accountability. Nurses felt many long-term patients already had well-established routines. Although most patients agreed, those who changed their routine, for example, dining out, found the text reminder was useful to them. Nevertheless, both patients and nurses believed that this intervention would be most useful for patients at commencement of drug therapy. Patients found discussing their medication side effects with the nurse and receiving expert advice regarding self-management highly beneficial. Other benefits included increasing awareness of self-care, encouraging informed decision making and feeling reassured.

The usability of the intervention was high: most patients expressed ease with text reminders and the weekly symptoms survey. A small proportion of patients did experience difficulty either receiving or responding to the text within 2 hours either because they were not used to carrying a cellular phone or slow Internet speed. However, as the adoption of cellular phones/devices continues to rise and Internet speed and geographic coverage improve [61], this barrier is likely to diminish in future.

The nurses endorsed all facets of the system, including the administration website, QPL, resource and intervention manuals, session checklists, symptom surveys, automatically generated advice, and nurse phone consultations. They suggested improving the layout of the intervention manual and less frequently emailed daily digest (or summary) of patient adherence and symptom responses. One departure from the intervention protocol was nurses not calling patients when patients were less than 90% adherent. It is recommended that the application of the protocol is routinely monitored and departures are addressed through ongoing supervision and training. In particular, the importance of contacting patients who are less than 90% adherent immediately, not waiting until the next scheduled consultation, should be emphasized. Supervision could include role playing these scenarios to increase the skills to deal with issue.

### Limitations

The limitations of this study include a relatively small sample of nurses and patients used to pilot test this intervention. Given the sample size, it is unlikely that saturation was reached. As CML is a relatively rare disease, it proved difficult and time-consuming to achieve this modest sample size. Furthermore, most patients had been diagnosed years before the study, hence possibly had higher levels of medication adherence than those newly diagnosed. Future trials may need to explore a different cancer type to test the impact of this intervention upon oral medication adherence. As high-cost oral therapeutics in oncology continue to become more prevalent, it will be critical to ensure that adherence is high to deliver the anticipated survival outcomes to ensure that the health care expenditure is justified [4].

### Comparison With Prior Work

Despite phone text reminder interventions for patients’ with HIV [37], cardiac disease [35], and other chronic conditions [38] demonstrating improvements to medication adherence, not all studies included nurse or counselor supportive care in the intervention. In our study, an innovative medication intervention was developed and tested, which combined 2 features: (1) daily medication reminders and routine assessment of side effects with evidence-based self-care advice delivered in real time; and (2) QPL questions and routinely collected individual patient adherence and side-effect profile data used to shape nurses’ consultations, which employed motivational interviewing to support adoption of self-management behaviors.

A secondary analysis [41] identified 2 randomized controlled trials of phone text reminder adherence interventions combined with counseling sessions, 1 study in patients with HIV [62,63] and the other in patients with diabetes, which has since been retracted. Qualitative findings indicated beneficial experiences where patients with HIV emphasized emotional and mental support from health care providers in addition to medical care [63]. A similar finding has been described in patients with tuberculosis who reported feeling cared for and thankful that health professionals were available to answer their questions while reminders instilled a sense of responsibility and personal accountability for their treatment [33]. Comparably, patients receiving phone symptom monitoring and nurse support while undergoing chemotherapy for cancer have reported feeling reassured [45]. Although previous studies have recognized text reminders prompt adherence in the home environment as well as on vacation [21,34], some patients believed reminders may be more useful earlier in diagnosis before habits being formed [34]. Many of these sentiments were also expressed by patients in our study.

Patients in our study reported technological difficulties and an absence of cellular phones, whereas additional reasons for not responding have been reported in the literature. These include problems with cellular phones, out-of-phone credit, the phone being switched off, the phone’s battery running out, and losing the cellular phone [25,33,63]. It will be difficult for health services to overcome these types of practical problems and they may have to be accepted as a limitation of this type of intervention.

### Conclusions

This paper contributes to the evolving knowledge base of how to develop an evidence-based, acceptable, and potentially effective complex intervention. We have provided examples of strategies that may assist researchers and clinicians in developing or trying out a new psychoeducational intervention. These strategies can enhance intervention methodological rigor for testing in a randomized controlled trial through standardized intervention content and training; and implementation considerations such as improved clinical acceptability through the codesign process. To our knowledge, this is the first cancer medication adherence intervention that integrates a mHealth platform with tailored phone consultations based on patient-provided questions, adherence rates, and side-effect profiles provided by nurses trained in motivational interviewing. The intervention was clinically feasible. Patients and nurses endorsed this intervention as acceptable and useful particularly for a newly diagnosed patient. The next step will be to conduct an appropriately powered randomized controlled trial targeting a wider range of cancer diagnoses that are currently managed by oral therapeutics to ensure adequate recruitment and assess broader applicability. This future research will focus on patients at diagnosis and assess adherence with an electronic pill monitoring device to examine short- and long-term adherence. In addition, because chronic illnesses are more prevalent among older citizens, many people will have 2 or more comorbidities requiring self-management with oral medications. Future iterations of the REMIND platform should cater for multiple medications for multiple comorbidities. Health care resource utilization and sustainability of the intervention should also be considered by integrating an economic evaluation into the randomized controlled trial.

## Abbreviations

AIDS: acquired immunodeficiency syndrome
CML: chronic myeloid leukemia
HIV: human immunodeficiency virus
mHealth: mobile health
MRC: Medical Research Council
QPL: question prompt list
TKI: tyrosine kinase inhibitor
